# Pre-travel health care attendance among migrant travellers visiting friends and relatives (VFR): a 10-year retrospective analysis

**DOI:** 10.1186/s12889-019-7722-0

**Published:** 2019-10-28

**Authors:** Pietro Ferrara, Cristina Masuet-Aumatell, Josep Maria Ramon-Torrell

**Affiliations:** 1 Department of Experimental Medicine, University of Campania “Luigi Vanvitelli”, 5 Via Luciano Armanni, 80138 Naples, Italy; 2 Bellvitge Biomedical Research Institute (IDIBELL), Preventive Medicine Department, University Hospital of Bellvitge, Feixa Llarga s/n, 08907 L’Hospitalet de Llobregat, Catalonia Spain; 3 Clinical Science Department, School of Medicine, University of Barcelona, Feixa Llarga s/n, 08907 L’Hospitalet de Llobregat, Catalonia Spain

**Keywords:** Healthcare attendance, Immigrants, Travel medicine, Travellers visiting friends and relatives, Vaccination

## Abstract

**Background:**

Travellers visiting friends and relatives (VFR) define a specific population of travellers exposed to higher risks for health and safety than tourists. The aim of this study was to assess differentials in pre-travel health care in VFR travellers compared to other travellers.

**Methods:**

A retrospective cohort study was performed including attendees of the Travel Medicine Clinic of the Hospital Universitari de Bellvitge, Barcelona, Spain, between January 2007 and December 2017.

**Results:**

Over the 10-year period, 47,022 subjects presented to the travel clinic for pre-travel health care, 13.7% of whom were VFR travellers. These showed higher rates of vaccination against yellow fever and meningococcus, but lower rates for hepatitis A, hepatitis B, influenza, rabies, cholera, polio, typhoid IM vaccine and tetanus vaccine boosters. Regarding malaria prevention measures, results highlighted that VFR travellers, when compared with tourists, were more likely to be prescribed with chemoprophylaxis, particularly with mefloquine, than with atovaquone/proguanil.

**Conclusions:**

Findings from this large-scale study indicated differences in vaccination rates and completion, as well as in chemoprophylaxis for malaria, between VFR and non-VFR travellers, fostering specific interventions for promoting adherence to pre-travel health advice among migrant travellers.

## Background

According to the United Nations, the number of international refugees and migrants worldwide has continued to grow rapidly in recent years, reaching 258 million in 2017, with a number of migrants as a percentage (14%) of the population residing in high-income countries [1]. As a consequence of this geographic dispersion through movement of people, migrants who return to their place of origin represent a large number of international travellers, also forecasted to increase. Indeed, the public health construct “visiting friends and relatives (VFR)” categorises travellers—often ethnically distinct from the majority population of the country of residence—who travel to lower-income countries for the purpose of visiting friends and relatives [2, 3].

VFR travellers are exposed to higher risk of travel-related health problems than other travellers. Reasons for this risk variance are many. They generally stay longer than tourists in the country of destination, go to rural and higher-risk areas, have close contact with the local population, as well as are subject to local social and cultural practices (such as consuming high-risk foods and water served by hosts, type of accommodation and so on) [4].

Again, despite being at considerable risk for their health and safety, VFR travellers showed lower levels use of pre-travel health services and less adherence to pre-travel advice. Drivers for not consulting health professional before travelling were the underestimation of risks, including a scares knowledge of the disease and the perception that the measures undertaken would be sufficient, the belief of being experienced travellers, as well as financial limitations of the migrant population [5]. Moreover, in this susceptible traveller population, assessing previous exposure and immunisation and vaccination status is complicated by several cultural and socioeconomic barriers [6–8].

Yet, to the best of our knowledge, the information focusing on pre-travel assessment of VFR travellers currently available is limited, with little or no epidemiological studies conducted on a large scale in Europe and over time [9]. A recent small-scale study on the use of pre-travel health among VFR travellers, conducted in the United States, highlighted differentials in the level of pre-travel care between VFR and non-VFR individuals and lower vaccination rates in the VFR population [10].

Therefore, this study aimed to describe differences in the usage of pre-travel health care among different traveller cohorts over a 10-year period.

## Methods

This retrospective cohort study was conducted at the Travel Medicine Clinic of the Hospital Universitari de Bellvitge, in Barcelona, Spain, including attendees in the travel clinic between January 2007 and December 2017. The clinic is part of the public Catalan Health Service, with the price for pre-travel visit and vaccine administration (one or different types of vaccines) established as per national policies, as well as with a full coverage of the prescribed antimalarial chemoprophylaxis.

Pre-travel counselling was provided by travel medicine specialists of the unit and included information and recommendations of safety and health travels, in accordance with U.S. Centres for Disease Control and Prevention (CDC) guidelines [4], comprising vaccine indications, food safety, diarrhoea prevention, and information about the implementation of interventions to avoid mosquito bites (such as mosquito nets, use of repellent, and appropriate clothes) and malaria chemoprophylaxis.

The Institutional Ethical Review Board approved the study protocol and granted approval for reviewing records for the purpose of the study. Confidentiality was maintained by omitting any personal identifying information from data collection.

From the travellers’ recording tool, the following data were extracted for each individual: age, sex, area of origin, travel destination, purpose of travel (VFR and non-VFR, such as tourism and leisure, business, etc.), vaccines administered and prescription for malaria chemoprophylaxis.

The medical records reported the administration and completion of the following routine or travel-related vaccines: hepatitis A, hepatitis B, typhoid fever, yellow fever, meningococcus, polio, rabies, cholera, influenza, measles-mumps-rubella (MMR), tetanus and diphtheria-tetanus-pertussis (DTPa). Attendees refusing a recommended vaccine were considered not vaccinated.

Data on malaria prevention measures recorded participants who were prescribed mefloquine 250 mg or atovaquone 250 mg and proguanil 100 mg. Dosage of the medications used for chemoprophylaxis followed the recommendation provided by the CDC. Travellers prescribed with mefloquine were instructed to begin chemoprophylaxis 2 weeks before travel to malarious areas, continue once a week during travel in malarious areas and for 4 weeks after their return. Atovaquone/proguanil was prescribed to be started 1–2 days before travel to malarious areas and taken daily while in the malarious areas and for 7 days after leaving the area [4].

Continuous variables were expressed as mean and standard deviation (SD); categorical variables were described as number and percentage. The Student’s *t*-test for independent samples was used to assess differences between means; the chi-squared (*χ*^2^) test was used to assess differences between categories. Univariate analyses were also conducted to determinate vaccine completion and anti-malarial prescription between VFR and non-VFR travellers. Subsequently, independent variables (age, sex and travel destination) were included in a multivariate logistic regression model to adjust for potential confounders and estimate the likelihood of vaccine completion and anti-malarial prescription for VFR travellers. To perform logistic regression analysis, those variables with a *p*-value equal or less than 0.25 were, therefore, considered for inclusion. A stepwise backward selection procedure was used, fixing a *p*-value of ≤0.40 as the criterion for removing variables from the final multivariate regression models. Results were reported as crude and adjusted odds ratios (ORs) and 95% confidence intervals (CIs). All statistical tests were two-tailed, and a *p*-value ≤0.01 was considered statistically significant. Data were analysed using Stata 14 statistical software [11].

## Results

### Study population

Over the 10-year period, 47,022 subjects attended to the Travel Medicine Clinic of the Hospital Universitari de Bellvitge for pre-travel health care, constituting the cohort included in this study. Approximately half (51.7%) were female, with a mean age of 34.7 years (± 13.9 SD). The vast majority of travellers were European (84.7%), with 39,102 travellers from Spain. Regarding travel information, the most popular destinations were Africa, South-eastern Asia and South America. VFR travellers represented 13.7% of the cohort, originating mainly from South America (49.9%) and Africa (31.7). Baseline characteristics of the study population are reported in comparative Table 1. Comparison between VFR and non-VFR cohorts revealed statistically significant differences above all investigated baseline characteristics (*p* <  0.001 for all).

**Table 1:** Baseline characteristics of study participants (*n* = 47,022)

	All (*n* = 47,022)	Non-VFR (*n* = 40,569)	VFR (*n* = 6453)	Comparison
	N (column %)	N (column %)	N (column %)	*P* value°
Sex				< 0.001
Male	22,712 (48.3)	20,224 (49.8)	2488 (38.6)	
Female	24,310 (51.7)	20,345 (50.2)	3965 (61.4)	
Age^a^	34.7 ± 13.9	35.8 ± 12.5	28.7 ± 18.7	< 0.001
Area of origin				< 0.001
Africa	2257 (4.8)	215 (0.5)	2042 (31.7)	
Asia	752 (1.6)	124 (0.3)	628 (9.7)	
Australia	19 (0.0)	12 (0.0)	6 (0.1)	
Europe	39,828 (84.7)	39,764 (98.0)	64 (1.0)	
North America	28 (0.1)	20 (0.1)	8 (0.1)	
Cen. America	517 (1.1)	36 (0.1)	481 (7.5)	
South America	3621 (7.7)	397 (1.0)	3223 (49.9)	
Travel destination				< 0.001
Africa	14,859 (31.6)	12,779 (31.5)	2080 (32.2)	
Asia	6301 (13.4)	5842 (14.4)	459 (7.1)	
SEA	11,614 (24.7)	11,481 (28.3)	133 (2.1)	
Cen. America	3950 (8.4)	3489 (8.6)	461 (7.1)	
South America	9640 (20.5)	6369 (15.7)	3270 (50.7)	
Others	658 (1.4)	609 (1.5)	49 (0.8)	

*VFR* Travellers visiting friends and relatives, *SEA* South-eastern Asia

°*P* value obtained by chi-squared (*χ*^2^) and Student’s *t*-tests

^a^Mean ± standard deviation

### Vaccinations

**Fig. 1:**
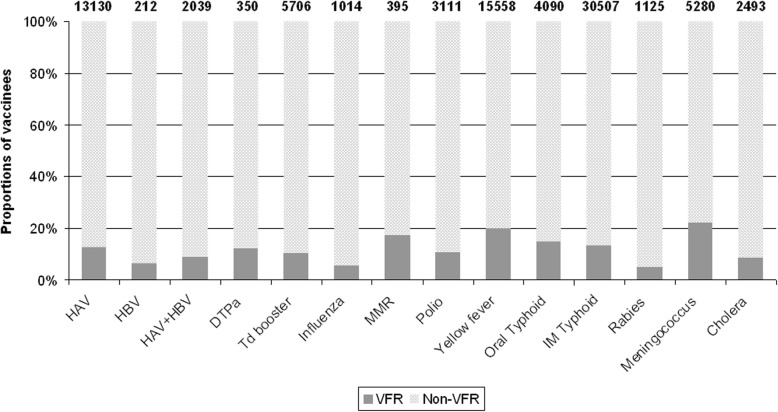
Total number of individuals administered each vaccine (first dose) and proportions of VFR and non-VFR vaccinees. *VFR,* travellers visit friends and relatives; *HAV*, hepatitis A; *HBV*, hepatitis B; *MMR*, measles-mumps-rubella; *DTPa*, diphtheria-tetanus-pertussis

Figure 1 shows the total number of travellers who were administered vaccines, and the proportions of VFR and non-VFR travellers. The analysis showed differences in vaccination completion between the two groups (Table 2). Except for yellow fever and meningococcus vaccines, where the odds of vaccination were about twice greater in the VFR population, non-VFR travellers showed higher rates of vaccination against hepatitis A, hepatitis B (first dose), influenza, rabies, cholera, polio, typhoid IM vaccine and tetanus vaccine booster.

**Table 2:** Rates of administration of vaccinations (*n* = 47,022)

	All (*n* = 47,022)	Non-VFR (*n* = 40,569)	VFR (*n* = 6453)		
	N (%)	N (%)	N (%)	cOR (95% CI)	aOR (95% CI)^a^
HAV					
1st dose	13,130 (27.9%)	11,469 (28.3)	1661 (25.7)	0.88 (0.83–0.93)	0.59 (0.55–0.64)
2nd dose	3024 (6.4%)	2732 (6.7)	292 (4.5)	0.66 (0.58–0.74)	0.56 (0.48–0.65)
HBV					
1st dose	212 (0.5%)	198 (0.5)	14 (0.2)	0.44 (0.26–0.76)	0.35 (0.18–0.70)
2nd dose	75 (0.2%)	67 (0.2)	8 (0.1)	0.75 (0.36–1.56)	0.99 (0.46–2.17)
3rd dose	34 (0.1%)	31 (0.1)	3 (0.1)	0.61 (0.19–1.99)	0.51 (0.12–2.18)
HAV + HBV					
1st dose	2039 (4.3%)	1852 (4.6)	187 (2.9)	0.62 (0.54–0.73)	0.61 (0.51–0.72)
2nd dose	442 (0.9%)	418 (1.0)	24 (0.4)	0.35 (0.24–0.54)	0.35 (0.21–0.61)
3rd dose	167 (0.4%)	150 (0.4)	17 (0.3)	0.71 (0.43–1.18)	0.73 (0.41–1.30)
Tetanus					
DTPa	350 (0.7%)	307 (0.8)	43 (0.7)	0.88 (0.64–1.21)	0.85 (0.58–1.24)
Td booster	5706 (12.1%)	5109 (12.6)	597 (9.3)	0.71 (0.65–0.77)	0.66 (0.59–0.73)
Influenza	1014 (2.2%)	955 (2.4)	59 (0.9)	0.38 (0.29–0.50)	0.43 (0.28–0.64)
MMR	395 (0.8%)	326 (0.8)	69 (1.1)	1.33 (1.03–1.73)	0.77 (0.52–1.13)
Polio	3111 (6.6%)	2775 (6.8)	336 (5.2)	0.75 (0.67–0.84)	0.70 (0.61–0.81)
Yellow fever	15,558 (33.1%)	12,439 (30.7)	3119 (48.3)	2.12 (2.01–2.23)	2.00 (1.88–2.13)
Typhoid					
Oral vaccine	4090 (8.7%)	3485 (8.6)	605 (9.4)	1.10 (1.01–1.20)	1.15 (1.00–1.29)
IM vaccine	30,507 (64.9%)	26,437 (65.2)	4070 (63.4)	0.91 (0.86–0.96)	0.79 (0.74–0.85)
Rabies					
1st dose	1125 (2.4%)	1069 (2.6)	56 (0.9)	0.32 (0.27–0.42)	0.22 (0.16–0.31)
2nd dose	88 (0.2%)	84 (0.2)	4 (0.1)	0.30 (0.11–0.81)	0.26 (0.08–0.86)
3rd dose	51 (0.1%)	51 (0.1)	0 (0)	–	–
Meningococcus	5280 (11.2%)	4110 (10.1)	1170 (18.1)	1.96 (1.83–2.11)	3.01 (2.72–3.33)
Cholera	2493 (5.3%)	2278 (5.6)	215 (3.3)	0.61 (0.53–0.71)	0.68 (0.58–0.80)

*VFR* Travellers visit friends and relatives, *HAV* Hepatitis A, *HBV* Hepatitis B, *MMR* Measles-mumps-rubella, *DTPa* Diphtheria-tetanus-pertussis, *cOR* Crude odds ratio, *aOR* Adjusted odds ratio, *CI* Confidence interval

^a^Adjusted for age, sex, and travel destination

No significance was found in rates of completion of oral typhoid, DTPa, MMR and of the entire series of hepatitis B vaccines, as well as when to vaccinate hepatitis A.

### Malaria prevention

Of the whole population, 16,255 were prescribed malaria chemoprophylaxis. In general, VFR travellers were more likely to be prescribed malaria chemoprophylaxis (43.5% vs. 33.1%, *p* <  0.001). Stratified data on type of medication prescribed for the prevention of malaria showed that, independent of travel destinations, non-VFR travellers had a better than 28% chance of receiving atovaquone/proguanil prescription, whereas VFR travellers were more likely to be prescribed mefloquine (Table 3).

**Table 3:** Travellers prescribed malaria chemoprophylaxis (*n* = 16,255)

Chemoprophylaxis	Non-VFR	VFR	cOR (95% CI)	aOR (95% CI)^a^
	N (%)	N (%)		
Mefloquine	2829 (17.4)	1724 (10.6)	4.86 (4.55–5.20)	6.21 (5.72–6.76)
Atovaquone/Proguanil	10,582 (65.1)	1120 (6.9)	0.60 (0.56–0.64)	0.72 (0.67–0.78)

*VFR* Travellers visiting friends and relatives, *cOR* Crude odds ratio, *aOR* Adjusted odds ratio, *CI* Confidence interval

^a^Adjusted by age, sex, and travel destination

## Discussion

This large-scale study, the first performed in Spain, offers a first important insight into differences in vaccination between VFR and non-VFR travellers.

It is important to remember that the VFR population differs from travellers other than VFR in sociodemographic characteristics. These travellers, in fact, were younger than non-VFR travellers; were for the most part women; and, as expected, composed mainly of people from non-European countries. The analysis of vaccination rates between the two cohorts indicated some differentials in adherence to immunisation protocols. First of all, it should be noted that the level of completion of those vaccines was not properly related to travel. Indeed, vaccines against influenza, polio, tetanus and both hepatitis A/B and hepatitis B were less likely to be completed by VFR travellers. Regarding hepatitis, noticeable is the scarce level of vaccine completion in the VFR travellers, since their most frequent destinations were in African countries, where hepatitis B is spreading on an endemic level. On this point, previous epidemiological research highlighted that a great percentage of hepatitis B in VFR travellers returning to their residency country are in those coming back from Africa (mainly, from sub-Saharan Africa), where the major epidemiological determinant for hepatitis B infections in VFR travellers is represented by the higher likelihood of sexual encounters with natives during travel [12]. However, a limitation exists because VFR travellers usually do not have a childhood immunisation card, so the prescription is provided during the visit or, if some period of time exists prior to travel, a serology is done to assess the need for immunisation. In Catalonia, travellers born after 1983 have been vaccinated against hepatitis B in adolescence, and all have a childhood immunisation card. Catalan travellers born before 1983 or immigrants without a registry about immunisation are usually prescribed hepatitis B if the endemicity is higher than that in our country.

Migration in Catalonia started at the beginning of the XXI century, so VFRs do not travel much more often than non-VFR travellers. They save money from their arrival to come back home as soon as possible, but it was difficult to travel once during the first decade, because flights were expensive [13]. Therefore, VFR and non-VFR adults, once they are seen at the travel clinic (in a real-life context), probably show that they have not received any vaccine since childhood, and no immunisation history is present, so no difference between them exists [14].

Low levels of immunisation for the other routine vaccines are likewise a worry; staying too long in rural areas, in close proximity to the local population, in suboptimal hygienic conditions, exposes travellers to diseases from which the population should be protected [2, 3]. In this regard, one of the most relevant challenges in the health care of migrants is represented by the difficulty of obtaining clear information on their immunisation status because of partial or no data regarding previous exposures or vaccinations [15]. This aspect, indeed, forms part of the wider problems of inequalities in access to health services in the countries where migrants live, likely due to sociocultural, economic (costs) and language barriers [16–18]. These are the same drivers that—together with others (such as immunisation hesitancy, low risks perception, limited time before the travel, characteristics of the travel and so on)—could probably lead VFR travellers to ignore pre-travel care and advice before they travel. On the other hand, this study can be considered a pragmatic or real-life study, because usually adults in Catalonia do not have an immunisation card, and neither do immigrants.

A constant source of concern about travellers’ health is food and water safety, with special attention to the prevention of traveller’s diarrhoea and other food-related illnesses [19]. VFR travellers are commonly exposed to these risks more than tourists, due to eating contaminated food (crude food, freshwater fish and so on), sharing meals with local hosts and limited access to water. The low level of adherence to hepatitis A, typhoid IM and cholera vaccines among VFR travellers calls attention to the potential implications for their health. One possible explanation for the lower vaccination rate against hepatitis A amongst VFR travellers is that this population might be exposed to the infection in their home-country during childhood, reducing the need for this specific protection. More research should deepen this aspect. However, typhoid fever or cholera should be emphasised in their vaccination regimen because of an increased risk of exposure in rural areas in endemic countries.

Moreover, travelling to remote areas necessarily entails a greater attention to specific risks and demands about immunisation. One of these is represented by the increased exposure to animal bites, even those kept by local families; then protection against rabies becomes important, especially for the high mortality rate of the disease [20]. In this regard, VFR travellers showed significantly less adherence to rabies vaccination than other travellers did.

Interesting, instead, is the finding that the odds of acquiring yellow fever vaccination were twice greater in the VFR population than among non-VFR travellers, partially in accordance with the results reported by Tan et al. [10] but displaying a lack of concordance if compared to published research conducted to assess travel characteristics about the adherence to yellow fever vaccination [21]. This might be explained by the fact that VFR travellers had planned travels to countries or areas endemic for yellow fever, and they probably are very conscious of the danger of the disease. Another reason that may lead VFR travellers to acquire this vaccine is that travellers are required to present a proof of vaccination to enter certain countries. Indeed, the requirement of a vaccination certificate may explain the higher rate of meningococcal vaccinations among VFR travellers.

Travel destinations and length of stay might influence the level of prescription of antimalarial drugs between the two groups. VFR individuals travelled more to malaria zones than tourists did, so they were prescribed personal protection measures against malaria. However, independent of travel destinations, VFR travellers received more mefloquine than atovaquone-proguanil, probably due to time duration, but we could not confirm this. In South America, neither VFR nor non-VFR travellers usually went to malaria-endemic areas, so we usually did not prescribe antimalarial chemoprophylaxis.

Further studies should highlight the level and drivers of adherence to malaria chemoprophylaxis among VFR travellers, though. For instance, a study by Wieten et al., which only included VFR travellers to West Africa, found that age was positively associated with the probability of receiving pre-travel advice, as well as was a significant factor associated with the behaviours of buying and starting chemoprophylaxis before departure [22]. However, it should be mentioned that the research on the contribution of age on the adherence to malaria prevention measures needs further study [23].

VFR travellers are recognised as a peculiar traveller population, characterised by special health needs [24]. The presented findings showed a relevant gap in pre-travel care, with alarming differences between non-VFR and VFR travellers, and when rates of vaccination were suboptimal in both groups. Hypothetically, several drivers could be referred to as determinants in those differences. First, VFR travellers could have biased attitudes and perceptions about travel-related health risks, particularly when they are living in a home country with a low gradient of epidemiologic risks that might falsely reassure them, also fostering the belief that they are already immune, thus leading to less use of precautions and fewer personal protection measures than tourists take [4]. On this point, it is also important to highlight that several barriers could affect the pre-travel consultation. Indeed, it constitutes a complex process and its effectiveness strongly depends on relationship between physician and traveller [25, 26]. These are often provided with too much information, which can be misunderstood when communication and language barriers are present, as in the case of VFR travellers.

Finally, it is possible to affirm that specific interventions are needed to promote adherence to pre-travel health advice among VFR travellers, pointing out health education and health promotion for this group population, to achieve an optimal level of protection against travel-related risks [27, 28].

This study may have some methodological limitations that are worthy of emphasis and that should be considered when interpreting the results. First, the cohort nature of the study does not make it possible to establish causal effects. In addition, the database information of the travel clinic attendees did not include data on previous vaccinations (usually not recorded or registered nor provided by travellers), comorbidities (usually travellers say that they do not have any medical condition, and we could not recover data about that) and characteristics other than those mentioned in the Methods section: the possibility that associations found may be explained by other confounders should be taken into account. Second, the study includes only subjects seeking medical advice in our unit and does not include patients seeking it elsewhere or not seeking it at all, so this cohort might be inadequate for determining all the predictors of vaccines and chemoprophylaxis acceptance. Further studies on this issue should be encouraged. However, cohort studies use broader inclusion criteria and fewer exclusion criteria than randomised studies do, making results more generalisable for clinical practice.

Prior to a traveller’s attendance, all travellers are informed about the costs, so when travellers say that they do not want to be vaccinated, they will also pay the medical visit, so it is never an economical reason for why they are not vaccinated. The reasons for not vaccinating are that the inoculations are not mandatory to enter to their destination countries, or they need to receive different or consecutive doses (for example, for rabies). They say that they cannot come again to the travel clinic due to work limitations to obtain a free day for a medical visit.

Despite these limitations, the strengths of this study were that the cohort was properly selected and constituted a large number of participants; the study design was appropriate and the methodology of the study was accurate, reducing problems of bias.

## Conclusions

Given the rise in the number of international migrants, a significant proportion of travellers currently constitute VFR travellers, and the proportion of subjects travelling outside their home country (to return to their country of origin) is expected to increase. Findings from this study described differences in pre-travel care received between VFR and non-VFR travellers, with the latter showing lower rates of completion of recommended vaccinations, in spite of their increased risk for travel-related health problems and diseases. More research is needed in order to investigate factors that may explain the found differences.

In this frame, the pre-travel care of VFR travellers must be taken as a growing challenge to travel and preventive medicine.

## Data Availability

Data and supporting materials associated with this study will be provided upon request by contacting the corresponding author.

## Abbreviations

CDC: U.S. Centres for Disease Control and Prevention
DTPa: Diphtheria-tetanus-pertussis
HAV: Hepatitis A
HBV: Hepatitis B
MMR: Measles-mumps-rubella
VFR: Travellers visiting friends and relatives
